# CASE REPORT Persistent Seromas in Abdominal Free Flap Donor Sites After Postmastectomy Breast Reconstruction Surgery: Case Reports and Literature Review

**Published:** 2013-06-03

**Authors:** Abtin Sadeghi, Charles Malata

**Affiliations:** ^a^School of Clinical Medicine, University of Cambridge; ^b^Department of Plastic and Reconstructive Surgery, Cambridge Breast Unit, Addenbrooke's Hospital, Cambridge University Hospitals NHS Foundation Trust, Cambridge, UK

## Abstract

**Objectives:** Donor site seroma formation is a common occurrence following abdominal free flap breast reconstructions. Although such seromas usually resolve spontaneously after a few weeks or months, we recently encountered 3 patients with abdominal seromas persisting for up to 2 years postoperatively. We therefore investigated possible predisposing factors in our patient group. **Methods:** Patients with persistent abdominal seromas, arbitrarily defined as present after 3 months following abdominal free flap harvest were identified. Their demographic characteristics, comorbidities, reconstruction details, frequency, and volume of abdominal aspirations were documented. **Results:** Three obese patients (Mean body mass index = 35) with an average age of 49 years bilaterally reconstructed with superior inferior epigastric artery or deep inferior epigastric artery flaps fitted the aforementioned criteria. Seroma aspirations commenced at 3 weeks and continued for a maximum of 26 months postoperatively. The average number of aspirations was 11 with a mean volume of 338 mL (range: 100-864 mL) per visit. The patients were aspirated either weekly or fortnightly depending on the speed of seroma reaccumulation and symptoms. All the 3 patients needed excision of the seroma sac to achieve permanent resolution. **Discussion and Conclusion:** In addition to their nuisance value (notably frequent aspirations and outpatient clinic visits), persistent seromas can cause significant morbidity and eventually require surgical excision. Possible predisposing factors in our patients included obesity, bilateral reconstructions, and superior inferior epigastric artery flap harvest. Such “high risk” patients should be warned about the likelihood of persistent seromas needing repeated aspirations and possible surgical interventions for ultimate resolution.

Seroma formation is a common postoperative occurrence at the donor site following abdominal flap breast reconstructions. Indeed, the incidence in reconstructive and aesthetic abdominal procedures such as panniculectomy, abdominoplasty, and abdominal flaps can range from 1% to 38%.^1^ Despite this high prevalence, postoperative seromas usually resolve spontaneously after a few weeks or months, usually requiring no more than a few aspirations. However, we have recently treated 3 patients whose seromas persisted for up to 2 years following abdominal flap breast reconstruction highlighting a hitherto poorly documented outcome in reconstructive breast surgery.

The reported risk factors for abdominal seroma formation include obesity,^2^ the use of superior inferior epigastric artery (SIEA) flaps (compared to deep inferior epigastric artery [DIEP] or transverse rectus abdominus myocutaneous [TRAM] flaps),^3^ wide tissue undermining, extensive cautery dissection, and large abdominal tissue excisions.^1^ The latter may be an indirect measure of the patient's body weight. The primary objective of the present report was to document the common traits among our patients with the specific aim of identifying any possible predisposing causes for the seroma persistency. This information might then be useful in devising possible remedies.

## METHODS

In all cases, flaps were harvested by electrocautery dissection (Force FX-8C, setting 30, fulgurate setting, Valleylab, Boulder, Colo) and hemostasis was achieved with a combination of monopolar and bipolar diathermy. No fat was left on the fascia to optimize flap vascularity. Where relevant repair of the split in the rectus sheath was performed using number 0 looped nylon and the abdominal wound was closed over 2 suction drains (Blake's drains, Ethicon, New Jersey) using 2/0 PDS for the Scarpa's fascia and 3/0 Monocryl for the deep dermal and subcuticular layers. For each patient, the total time the seroma was present, the number of aspirations, and the average and total aspirate volumes are given in the Table 1.

The drain protocol used by the senior author involves the insertion of 2 suction drains (Blake's drains) kept under vacuum and the output recorded once every 24 hours at midnight. If the drain output is more than can be contained by the drain bag, this is emptied once every nursing shift, that is, 8 hours. The drains were removed once output had fallen to less than 30 mL in 24 hours (13 days in all the 3 patients).

## RESULTS

The patient details are summarized in the Table. None of the patients were smokers or diabetic and all of them underwent bilateral microvascular breast reconstructions with abdominal flaps as detailed in the Table.

**Figure 1:**
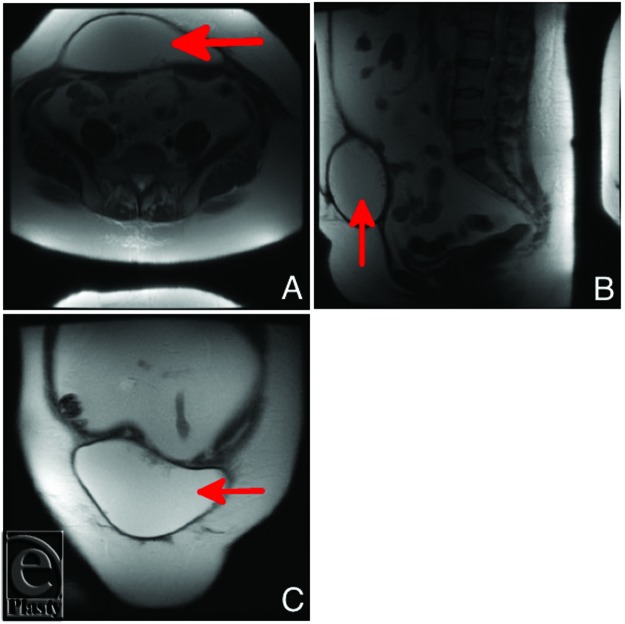
MRI scan of a 50-year-old patient done 21 months after immediate breast reconstruction. The patient had previously undergone excision of a seroma sac (“capsulectomy”) 12 months prior to the imaging. The scan shows a high signal intensity, nonloculated lesion on T2W measuring approximately 19 × 7 × 11 cm^3^ in the subcutaneous tissues immediately anterior to the rectus muscles. (*a*) Transverse view, (*b*) Sagittal view, and (*c*) Recumbent view showing the size of the seroma and its location superficial to the abdominal musculature. MRI indicates magnetic resonance imaging.

A 50-year-old woman (BMI = 32) with Cowden's syndrome and hypothyroidism underwent prophylactic mastectomies and immediate microvascular breast reconstruction with a left SIEA and right DIEP flap. There were no early postoperative complications and her first seroma aspiration was 2 weeks postoperatively. Her seroma history postreconstruction extended to 26 months during which time she needed seroma cavity washouts under general anesthetic, debridement, 2 drain insertions under ultrasound guidance, and 2 seroma sac “capsulectomies” (Fig 1). During this period, her seromas resulted in anemia (requiring a blood transfusion during surgery) and 2 emergency admissions, once from an expanding abdominal hematoma and once from an infected seroma cavity. Her total additional hospitalization was 25 days.

**Figure 2:**
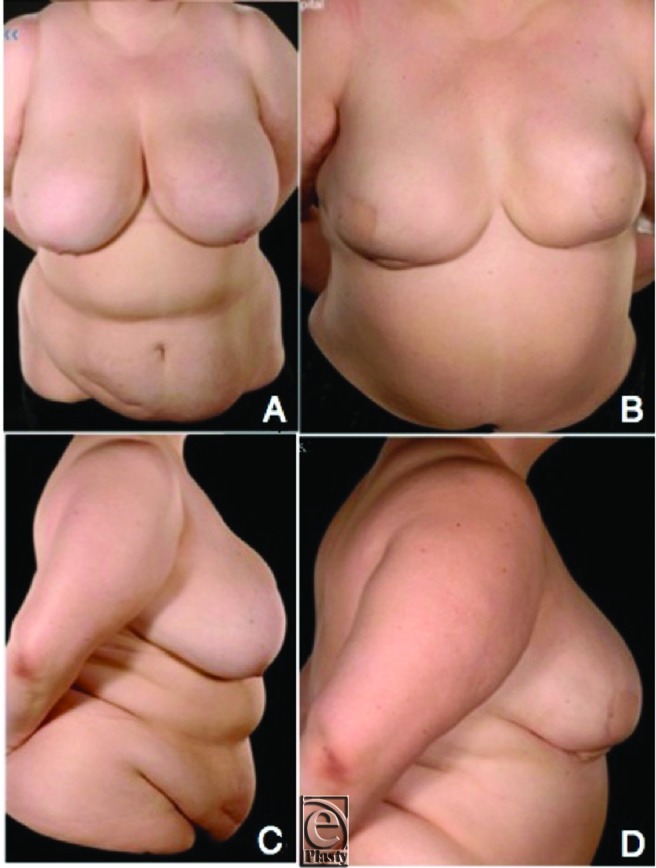
Preoperative images of patient 2 (BMI = 40.8), with a previous appendectomy and hysterectomy (*a* and *c*). Appearances after bilateral prophylactic mastectomies and immediate bilateral SIEA flap breast reconstruction (*b* and *d*). BMI denotes body mass index; SIEA = superior inferior epigastric artery.

A 43-year-old woman (BMI = 40.8) with a previous history of ovarian cancer and rheumatoid arthritis underwent immediate bilateral SIEA flap breast reconstructions (Fig 2) with an excellent early postoperative recovery. Seroma aspirations started 2 weeks after surgery and went on for 6 months. During this time, she required ultrasound-guided drain insertion (drains in situ for 2 weeks) and finally a “capsulectomy” before seroma formation was finally controlled. She also suffered from bleeding into her seroma cavity. Her total additional hospitalization amounted to 11 days.

A 54-year-old hypertensive (BMI = 32.1) underwent a right immediate and left delayed bilateral DIEP flap breast reconstruction. She had an uneventful recovery from surgery, but she required seroma aspirations commencing 3 weeks postoperatively, which continued until 6 months. In addition she underwent excision of her seroma cavity and drain insertion.

## DISCUSSION

### Risk factors

Seroma formation is a common donor-site sequel after abdominal flap breast reconstruction, with reported figures of 2% to 10%.^5^ However, in practice, this is higher as seroma is often considered a minor complication and is thus underreported by patients and surgeons alike.^5^ Clinically most seromas last only a few weeks and either resolve spontaneously or by aspiration. The data are sparse for abdominal tissue breast reconstruction. Kim and Stevenson showed that cosmetic abdominoplasty seromas resolved after a mean of 2.5 aspirations.^2^ In fact, it has recently been shown that the rate of seroma in DIEP flap surgery is even lower than abdominoplasy.^6^ In contrast, our patients required 3 to 17 aspirations (some of these following seroma sac excision), and their seromas persisted for an average of 12.7 months (range = 6–26 months). Several factors may have influenced this outcome.

The most frequently reported predisposing factors for seroma formation after the harvest of abdominal tissue are obesity and the use of SIEA flaps.^4^^,^^3^ Another important risk factor is bilateral autologous tissue breast reconstructions, most probably because these patients usually have high BMIs and are prone to donor-site problems.^4^ All our patients were obese, they underwent bilateral reconstructions, and 2 of them had SIEA flaps. Although SIEA flaps are the quickest and least invasive method of obtaining an abdominal donor flap for breast reconstruction in patients with compatible anatomy,^7^ SIEA flaps have been associated with an increase in inpatient abdominal drain volume relative to DIEP flaps.^3^ There are no reports in the literature of the persistence of seromas beyond the early postoperative period of up to 6 weeks. Two of our 3 patients had undergone a SIEA flap, and a study comparing seroma persistence in DIEP and SIEA flap patients would be useful in determining whether this is an associated factor or not.

Raised BMI has been associated with an increased rate of donor-site complications following autologous microsurgical breast reconstruction.^4^ It is also a well-documented significant risk factor for seroma formation in latissimus dorsi flap breast reconstruction.^8^ The 3 patients in the present report were clinically obese (BMI **≥**30) and would thus all be at an increased risk. It is also interesting that all our patients had bilateral breast reconstructions although this is not directly related to the abdominal donor site as the volume or quantity of tissue harvested is the same in bilateral and unilateral breast reconstructions albeit that only part of the resultant abdominal flap is used for the latter. It may be that bilaterality is merely a proxy for the size of the patient (high BMI).

### Effects of persistent seromas on patient outcome

Persistent seromas create many problems for the patient and the healthcare system. First, they are a nuisance, both in terms of the patient having to take time off work and spend money on travel to and from the hospital; and in terms of using up valuable outpatient and inpatient hospital resources. As discussed earlier, these patients have repeated aspirations resulting in frequent outpatient clinic or dressing clinic attendances.

Second, if left untreated, seromas may lead to the formation of a “capsule” (the so-called pseudobursa), thus creating a secondary problem that must be treated surgically.^9^ Repeated skin puncturing for seroma aspiration may also lead to an infection of the fluid or surrounding tissues as happened in one of our patients following ultrasound-guided seroma fluid aspiration. Needle puncture can also cause bleeding into the seroma cavity, which was seen in 2 of the 3 patients; and in one such patient, repeated bleeding led to anemia needing a blood transfusion. These episodes of bleeding occurred spontaneously but in one case resulted from a traumatic fall onto the abdomen. Like anemia, repeated aspirations can lead to hypoproteinemia (because of loss of protein in the seroma fluid) with its consequent problems.

### Prevention and treatment

These 3 cases highlight the possibility of seroma persistence for an inordinate amount of time and document possible complications. Thus where possible, patients at a high risk of forming persistent seromas should be counselled in how best to reduce their chances, for example, by reducing their BMI if this is feasible in the context of their cancer treatment. In addition, preventive measures such as the use of quilting sutures should be actively considered in such patients as the long-term benefits outweigh the immediate costs. Quilting reduces the incidence of seromas by minimizing the dead space intra- or postoperatively.^11^ This is important as emphasized by Matarasso et al^12^ and has been shown to be effective in Latissimus Dorsi flaps.^13^ However, quilting was not found to decrease seroma formation in TRAM flaps.^14^ Another technique for achieving this objective is to leave surgical drains in for a longer period, as is routinely done in the United States. However, quilting sutures have been shown to be most effective in abdominoplasty.^15^ Fibrin sealant has also been used for this.^16^ Other active interventions for the treatment of seroma could be undertaken earlier. Intraseromal steroid injections, useful in reducing reaccumulation of seroma in extended latissimus dorsi flaps,^10^ may present a simple, low cost, and safe method for reducing seroma incidence. In high-risk patients, it may be useful to undertake early “capsulectomy” (excision of the seroma sac), as these tend to be recalcitrant and promote seroma persistence.

## CONCLUSION

Persistent seromas after abdominal flap breast reconstruction are not only a nuisance to the patient but also occasionally require medical or surgical intervention to treat them. They also have cost implications for the healthcare provider. While the present small case series raises a number of interesting issues, a larger, preferably prospective, study is required to better understand the risk factors for persistent abdominal seromas and the effects of potential interventions.

## Figures and Tables

**Table 1:** Reconstructions and seroma details

Pt	Flap Type	Weight	Time between surgery & last aspiration	Mean (range) Aspirate (ml)	Total Aspirate (ml)	Number of Aspirations	Additional Hospital stay (days)
1.	R SIEA	1644g	26 months	320.8 (100-800)	5455	17	25
	L DIEP	1688g					
2.	R SIEA	1508g	6 months	519.9 (100-864)	6759	13	11
	L SIEA	1738g					
3.	R DIEP	1252g	6 months	310 (200-500)	930	3	5
	L DIEP	NS					

Pt = patient, NS = not specified
